# Efficacy of Clopidrogel on Reperfusion and High-Sensitivity C-Reactive Protein in Patients with Acute Myocardial Infarction

**DOI:** 10.1155/2009/932515

**Published:** 2009-04-12

**Authors:** Mehmet Akbulut, Makbule Kutlu, Yılmaz Ozbay, Veli Polat, Mehmet Nail Bilen, Adil Baydas, Yakup Altas

**Affiliations:** ^1^Department of Cardiology, Faculty of Medicine, Fırat University, 23100 Elazığ, Turkey; ^2^Cardiology Clinic, Harput State Hospital, 23110 Elazığ, Turkey

## Abstract

We investigated the effects of clopidogrel on reperfusion and inflammatory process in STEMI. A total of 175 STEMI patients with similar clinical characteristics were included to this study. One was the standard pharmacological reperfusion therapy group (group 1, *n* : 90), who received 300 mg aspirin, 70 U/kg bolus, and 12 U/kg/hr continuous infusion of unfractioned heparin and accelerated t-PA. Clopidogrel 450 mg loading and 75 mg/d thereafter was added to standard reperfusion therapy in the other group (group 2, *n* : 85). The ST-segment resolution, CK-MB, and high-sensitive CRP (hs-CRP) parameters were measured. Complete ST resolution was observed in 32 patients (36.8%) in group 1 and 53 patients (63.8%) in group 2 (*P* < .001). Also in the first 24 hours, the CK-MB levels of patients in group 1 were significantly higher than those of group 2 (*P* = .001). The hs-CRP values were greater in group 1 than group 2 at 48th hour (gruop 1: 9.4 ± 0.1 mg/L, group 2: 3.7 ± 1.4 mg/L; *P* = .000). We concluded that adding clopidogrel to standard treatment in STEMI patients provided early reperfusion and suppression of inflammatory response.

## 1. Introduction

 Thromboxane and ADP, which are among the mediators of platelet activation and aggregation, play a key role in initiating and propagating coronary thrombosis and are raised during myocardial infarction. Simultaneous inhibition of both of these pathways with the combination of clopidogrel and aspirin produces greater antiplatelet effects than either agent alone [1, 2]. In recent reports, dual antiplatelet therapy with aspirin and clopidogrel showed significant improvements on prognosis in ST-elevating myocardial infarction (STEMI) [3–5]. Moreover, this beneficial effect is not only limited to the acute phase, but also extended to a 1-year follow-up period [6]. But substantial uncertainty remained regarding the net effects of adding clopidogrel to aspirin upon the reperfusion, acute inflammatory response, and ischemic events in this setting. In this study, we evaluated the effect of the addition of clopidogrel to standard reperfusion protocol on reperfusion and acute inflammatory response.

## 2. Material and Methods

### 2.1. Patient Population

 From December 2006 to January 2008, consecutive patients were enrolled who presented with ischemic discomfort lasting >20 minutes at rest within 12 hours, before randomization pain lasting >20 minutes, ST-segment elevation of at least 0.2 mV in at least two contiguous precordial leads. The study was limited to only anteriorly located myocardial infarction to homogenize the investigated population. The exclusion criteria were conduction or rhythm abnormalities (bundle branch block, idioventricular rhythm, etc.); any contraindication to thrombolytics [7]; early coronary angiography (within first 48 hours) due to recurrent ischemia or failed thrombolysis; those patients under the treatment of aspirin or thienopyridines; any contraindication to aspirin or clopidogrel; past history of MI or coronary revascularization; presence of clinically assessed heart failure (Killip II/III) or cardiogenic shock; hepatic failure; renal failure (serum creatinine >2.5 mg/dL); thrombocytopenia (<100.000/mm^3^) and patients older than 70 years old were excluded.

The study protocol was approved by institutional review board, and written informed consent was taken from all patients.

### 2.2. Study Design

A total of 175 patients were included in the present study and were allocated in 2 groups. One was the standard pharmacological reperfusion therapy group (group 1, *n* : 90), consisting of patients who received 300 mg aspirin at first then 150 mg/d thereafter, 70 U/kg (maximum 5000 U) bolus, and 12 U/kg/hr continuous infusion of unfractioned heparin and accelerated t-PA therapy (15 mg intravenous bolus followed by an infusion of 0.75 mg/kg (maximum 50 mg) over 30 minutes, followed by an infusion of 0.5 mg/kg (maximum 35 mg) over 60 minutes). Clopidogrel 450 mg loading and 75 mg/d thereafter was added to standard reperfusion therapy in the other group (group 2, *n* : 85). The ST-segment resolution within 120 minutes, CK-MB parameters within first 24 hours, and high-sensitive C-reactive protein (hs-CRP) parameters within 48 hours were measured. Also patients were examined for inhospital major and minor bleeding complications. All data were evaluated by two investigators who are blinded to treatment.

#### 2.2.1. Electrocardiographic Analysis

Standard 12-lead electrocardiograms were obtained at baseline and at 30-minute periods during the first120 minutes after initiation of fibrinolytic therapy. ST-segment elevation was analyzed manually with lens-intensified calipers to the nearest of 0.025 mV 20 milliseconds after the end of QRS complex with the PR segment as the reference baseline from leads I, aVL, and V_1_ through V_6_ for anterior infarction. The sum of ST deviation was measured at baseline and at 30, 60, 90, 120 minutes using previously described methods [8]. The percent resolution of ST deviation from baseline to 30, 60, 90, 120 minutes was calculated and categorized as complete (≥70%), partial (30% to 70%), or no resolution (≤30%).

#### 2.2.2. Blood Sampling

Blood samples were obtained for CK-MB and hs-CRP analysis at 0, 4, 8, 12, 24 and 0, 24, 48 hours of admission, respectively. Biochemical and haematological parameters were measured by an Olympus AU 600 autoanalyzer (Olympus Optical Co., Ltd., Schimatsu-Mishima, Japan) and Bayer Advia 120 Cell CBC Counter Hematologia autoanalyzer (Bayer Advia 120 CBC counter, NJ, USA). Hs-C-reactive protein was measured by the high-sensitivity nephelometric method (Dade Behring, Marburg, Germany).

### 2.3. Statistical Analysis

The SPSS for windows program (version 15.0) was used for all data analysis. Study data were expressed as mean value ± standard deviation or percent values. Numerical variables were compared by Mann-Whiney *U*-test, whereas nonnumerical ones were compared by Chi-squere test. Results with a *P* value less than .05 were considered statistically significant.

## 3. Results

### 3.1. Baseline Characteristics

A total of 175 patients were included in the study (group 1, *n* : 90; group 2, *n* : 85). Three patients due to ischemic events in group 1 (2 failed thrombolysis, 1 heart failure) and patients in group 2 were excluded from the study due to ischemic and hemorrhagic events (1 heart failure, 1 gluteal hemorrhage). However, there were no important differences in baseline clinical characteristics and initial therapy between the two treatment groups. Clinical characteristics of the study groups were shown in Table 1.

### 3.2. ST-Segment

**Figure 1:**
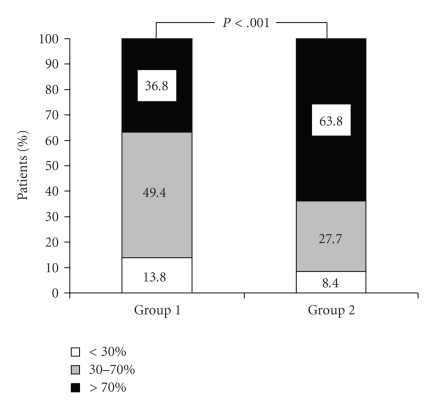
120 minute ST-segment resolution.

**Figure 2:**
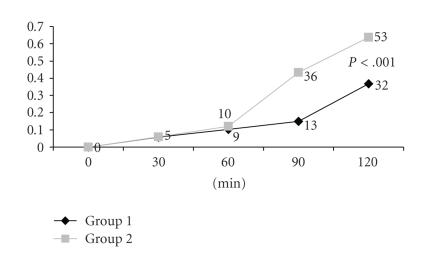
Patient number with ST-segment resolution in the course of time.

Complete ST resolution was achieved in 32 of patients (36.8%) in group 1 and 53 of patients (63.8%) in group 2 (*P* < .001) at the end of 120 minutes (Table 2 and Figure 1). The difference between groups started after 60th minute of the therapy, and it became more significant at 120th minute (Figure 2).

### 3.3. CK-MB

Peak CK-MB values were reached at 10–12th hours in group 1, whereas in group 2 they were at 8–10th hours. Peak CK-MB value was 233.5 ± 145 U/L in group 1 and 132.5 ± 122 U/L in group 2 (*P* = .01), and at the end of 24 hours CK-MB value was 138 ± 88 U/L in group 1, and 56 ± 35 U/L in group 2 (*P* = .001) (Table 3).

### 3.4. The hs-CRP

**Figure 3:**
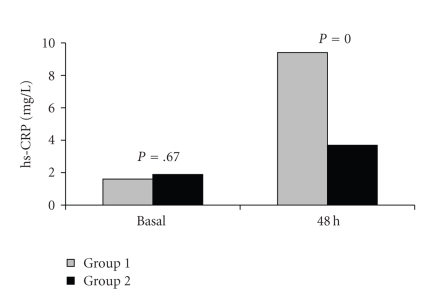
hs-CRP levels of the patients in the groups during the first 48 hours.

Although baseline hs-CRP values were similar at baseline (group 1: 1.6 ± 0.9 mg/L, group 2: 1.9 ± 1.3 mg/L; *P* = .67), they were greater in group 1 than group 2 at 48th hour (group 1: 9.4 ± 0.1 mg/L, group 2: 3.7 ± 1.4 mg/L; *P* = .000) (Figure 3).

### 3.5. Other Clinical Outcome

No death was observed in both groups. Failed thrombolysis in two patients and heart failure in one patient were seen in group 1; one patient with heart failure and one patient with major hemorrhage (≥2 unit blood transfusion) were seen in group 2. However, there is no significant difference between the groups with respect to these unfavorable events (*P* > .05).

## 4. Discussion

Platelet activation and aggregation play a key role initiating and propagating coronary artery thrombosis, and antiplatelet therapy improves outcomes across the spectrum of acute coronary syndromes. Beside this, it was thought that simultaneous inhibition of platelet activation and aggregation pathways with specific antiplatelet combinations might be more efficient in acute coronary syndromes [1, 2]. We observed improvements in both reperfusion and inflammatory response with the early addition of clopidogrel to standard reperfusion therapy in STEMI patients in this study. Moreover, these findings supported the hypothesis of dual antiplatelet inhibition with aspirin and clopidogrel might be more beneficial in acute coronary syndrome.

Currently, there are promising developments in the prevention of ischemic complications in sympthomatic atherothrombotic cases. The CLARITY-TIMI 28 study demonstrated that the addition of clopidogrel to aspirin in patients with STEMI receiving fibrinolytic therapy improved the patency rate of the infarct-related artery after 48 hours and reduced ischemic complications [3]. Among the patients in this trial who underwent percutaneous coronary intervention (PCI), described in the PCI-CLARTY substudy, clopidogrel pretreatment significantly reduced the incidence of cardiac death or ischemic complications both before and after PCI [9]. In the COMMIT trial, the routine addition of clopidogrel 75 mg/d to aspirin therapy for 4 weeks in patients with acute myocardial infarction resulted in a 9% proportional reduction in death, reinfarction, or stroke (*P* = .002) and a 7% proportional reduction in death (*P* = .03) [5]. Even though no loading dose was used, the benefit of clopidogrel therapy was seen almost immediately by an 11% proportional reduction in mortality in the first 2 days after the initiation of therapy (*P* = .019) [5]. The results of both CLARITY-TIMI 28 and COMMIT showed that adding clopidogrel to aspirin and other standard treatments safely reduced mortality and major vascular events in patients with acute myocardial infarction.

Beneficial effects of clopidogrel may arise through some different proposed mechanisms. (i) Clopidogrel may facilitate initial fibrinolysis and thereby improve early reperfusion with a glycoprotein IIb-IIIa inhibitor [10]-like effect. (ii) Clopidogrel may exert its effects in myocardial infarction mainly by preventing reocclusion or by limiting microvascular effects of platelet activation rather than enhancing fibrinolysis [5]. While most of the benefit of clopidogrel has been attributed to inhibition of platelet activation, blockage of the P2Y12 receptor may also have pleotropic effects with respect to endothelial cell function, leukocyte activation, and inflammation [11].

Previous studies have suggested some anti-inflammatory properties of clopidogrel. A reduction in the number of platelet-leukocyte interactions has been described [12, 13], and one study reported a special benefit of the drug in reducing the augmented risk of percutaneous interventions in patients with elevated levels of C-reactive protein [14]. In another study, it was stated that clopidogrel pretreatment attenuated the periprocedural increase in CRP by 65% [15]. Quinn et al. said that clopidogrel pretreatment reduces platelet inflammatory marker expression in patients undergoing PCI [16], and they also concluded that it might be very important for clinical events. All these findings constituted an important clue for us to investigate the limiting effect of clopidogrel in increment of high-sensitive C-reactive protein in STEMI and to verify this. Moreover, we could not see another study in literature about this topic.

Activated platelets have been clearly implicated in the tendency for microembolization during acute coronary syndrome [17, 18] and play an important role in the inflammatory response. Studies have shown that activated platelets express the CD40 ligand, a potent stimulus of vascular inflammation [19, 20]. The CD40 ligand promotes platelet-leukocyte interactions [19] and induces tissue factor expression [21], thus providing a link among inflammation, platelet activation, embolization, and coagulation. The inhibition of subcellular platelet CD40 ligand by clopidogrel treatment may explain the mechanism of reduction in inflammation as expressed by an attenuation in C-reactive protein increase after acute STEMI. By the way, we think that large extensive clinical trials are needed to exactly clarify the mechanisms of beneficial effects of clopidogrel on clinical results in previous ones and that is what we have determined in our study because the impressive improvement in clinical results with added clopidogrel on aspirin requires some other mechanisms except for a potent antiplatelet effect.

## 5. Limitations

The first and most important limitation is the small study population and the lack of a placebo control group. Only the anteriorly located STEMI patients were included to homogenize the study population, and a fibrinolytic agent only rt-PA was used and this condition was an important limiting factor for our study. Clopidogrel loading dose (450 mg) may be a limitation. We think that different dosages (300–600 mg) should be applied for both evaluations of safety and efficacy because there is a correlation between loading dose and time to reach maximal antiplatelet effect [22]. Another limiting factor of our study was the restriction of the study with 48 hours. A long follow-up period would be more useful to exactly realize all the efficiency and safety of clopidogrel (major and minor hemorrhages included) treatment. So that with long follow-up period, difference among the groups of medical treatment, PCI and coronary artery bypass grefting in the direction of angiographic findings could be clearly understood.

## 6. Conclusions

In conclusion, we found that addition of clopidogrel to medical reperfusion therapy in STEMI had favorable effects on reperfusion and suppression of hs-CRP. We think that these favorable clinical and laboratory effects of dual antiplatelet therapy with clopidogrel are not only limited to its antiplatelet effect, but also there may be some pleiotropic effects of clopidogrel; those were not explained clearly. Large extensive studies are needed to explain the complete effect of clopidogrel in acute STEMI.

## Figures and Tables

**Table 1:** Baseline characteristics.

Characteristics	Group 1 (*n* : 87)	Group 2 (*n* : 83)	*P* value
Mean age (year)	57 ± 7	56 ± 5	.31
Men (%)	79.3	80.7	.48
Time for symptom to lytic (h)	2.7 ± 2	2.6 ± 1	.56
Presentation			
* *Heart rate (beat/min)	75 ± 14	74 ± 12	.37
* *Systolic blood pressure (mmHg)	133 ± 14	135 ± 16	.31
* *Diastolic blood pressure (mmHg)	73 ± 11	75 ± 15	.29
History (%)			
* *Current smoker	44.8	44.6	.51
* *Diabetes mellitus	17.2	19.3	.44
* *Hypertension	29.9	32.5	.35
* *Hyperlipidemia	27.6	31.3	.35
High TIMI risk score (≥5)	13.8	14.8	.33
Medication during hospitalization (%)			
* * *β*-blocker	93	94	.50
* *Statins	90	93	.51
* *ACE-I or ARB	73	74	.43

**Table 2:** Number of patients with resolution in 120th minute.

	Group 1 (*n* : 87)	Group 2 (*n* : 83)	*P* value
>70%	36.8 (32)	63.8 (53)	.001
30–70%	49.4 (43)	27.7 (23)	.001
<30%	13.8 (12)	8.4 (7)	.01

**Table 3:** CK-MB (U/L) levels of the patients in the groups during the first 24 hours.

	0 hour	4th hour	8th hour	12th hour	24th hour
Group I	29.2 ± 23	106.4 ± 54	193.2 ± 125	233.5 ± 145	138 ± 88
Group II	28.5 ± 22	55.4 ± 35	132.5 ± 122	111.3 ± 27	56 ± 35
*P* value	.66	.006	.01	.001	.001

## References

[1] Gregorini L, Marco J (1997). Ticlopidine and aspirin interactions. *Heart*.

[2] Mishkel GJ, Aguirre FV, Ligon RW, Rocha-Singh KJ, Lucore CL (1999). Clopidogrel as adjunctive antiplatelet therapy during coronary stenting. *Journal of the American College of Cardiology*.

[3] Sabatine MS, Cannon CP, Gibson CM (2005). Addition of clopidogrel to aspirin and fibrinolytic therapy for myocardial infarction with ST-segment elevation. *The New England Journal of Medicine*.

[4] Sabatine MS, Morrow DA, Montalescot G (2005). Angiographic and clinical outcomes in patients receiving low-molecular-weight heparin versus unfractionated heparin in ST-elevation myocardial infarction treated with fibrinolytics in the CLARITY-TIMI 28 trial. *Circulation*.

[5] COMMIT (ClOpidogrel and Metoprolol in Myocardial Infarction Trial) collaborative group (2005). Addition of clopidogrel to aspirin in 45 852 patients with acute myocardial infarction: randomised placebo-controlled trial. *The Lancet*.

[6] Zeymer U, Gitt AK, Jünger C (2006). Effect of clopidogrel on 1-year mortality in hospital survivors of acute ST-segment elevation myocardial infarction in clinical practice. *European Heart Journal*.

[7] Antman EM (2008). ST-elevation myocardial infarction: management. *Braunwald's Heart Disease: A Textbook of Cardiovascular Medicine*.

[8] de Lemos JA, Antman EM, Giugliano RP (2000). St-segment resolution and infarct-related artery patency and flow after thrombolytic therapy. *The American Journal of Cardiology*.

[9] Sabatine MS, Cannon CP, Gibson CM (2005). Effect of clopidogrel pretreatment before percutaneous coronary intervention in patients with ST-elevation myocardial infarction treated with fibrinolytics: the PCI-CLARITY study. *The Journal of the American Medical Association*.

[10] Antman EM, Giugliano RP, Gibson CM (1999). Abciximab facilitates the rate and extent of thrombolysis: results of the thrombolysis in myocardial infarction (TIMI) 14 trial. *Circulation*.

[11] Quinn MJ, Plow EF, Topol EJ (2002). Platelet glycoprotein IIb/IIIa inhibitors: recognition of a two-edged sword?. *Circulation*.

[12] Storey RF, Judge HM, Wilcox RG, Heptinstall S (2002). Inhibition of ADP-induced P-selectin expression and platelet-leukocyte conjugate formation by clopidogrel and the P2Y12 receptor antagonist AR-C69931MX but not aspirin. *Thrombosis and Haemostasis*.

[13] Klinkhardt U, Graff J, Harder S (2002). Clopidogrel, but not abciximab, reduces platelet leukocyte conjugates and P-selectin expression in a human ex vivo in vitro model. *Clinical Pharmacology & Therapeutics*.

[14] Chew DP, Bhatt DL, Robbins MA (2001). Effect of *clopidogrel* added to aspirin before percutaneous coronary intervention on the risk associated with C-reactive protein. *The American Journal of Cardiology*.

[15] Vivekananthan DP, Bhatt DL, Chew DP (2004). Effect of *clopidogrel* pretreatment on periprocedural rise in C-reactive protein after percutaneous coronary intervention. *The American Journal of Cardiology*.

[16] Quinn MJ, Bhatt DL, Zidar F (2004). Effect of *clopidogrel* pretreatment on inflammatory marker expression in patients undergoing percutaneous coronary intervention. *The American Journal of Cardiology*.

[17] Davies MJ, Thomas AC, Knapman PA, Hangartner JR (1986). Intramyocardial platelet aggregation in patients with unstable angina suffering sudden ischemic cardiac death. *Circulation*.

[18] Frink RJ, Rooney PA, Trowbridge JO, Rose JP (1988). Coronary thrombosis and platelet/fibrin microemboli in death associated with acute myocardial infarction. *British Heart Journal*.

[19] Henn V, Slupsky JR, Gräfe M (1998). CD40 ligand on activated platelets triggers an inflammatory reaction of endothelial cells. *Nature*.

[20] Bhatt DL, Topol EJ (2003). Scientific and therapeutic advances in antiplatelet therapy. *Nature Reviews Drug Discovery*.

[21] Mach F, Schönbeck U, Bonnefoy J-Y, Pober JS, Libby P (1997). Activation of monocyte/macrophage functions related to acute atheroma complication by ligation of CD40: induction of collagenase, stromelysin, and tissue factor. *Circulation*.

[22] Popma JJ, Baim DS, Resnic FS (2008). Percutaneous coronary and valvular intervention. *Braunwald's Heart Disease: A Textbook of Cardiovascular Medicine*.

